# Endoscopic Endonasal Approaches to Anterior Cranial Base Meningiomas: Indications, Limitations, and Surgical Decision-Making

**DOI:** 10.3390/cancers18193217

**Published:** 2026-10-06

**Authors:** Manat Chopra, David John, David J. Cote, Gabriel Zada

**Affiliations:** Department of Neurological Surgery, Keck School of Medicine of the University of Southern California, Los Angeles, CA 90033, USA; dajohn@usc.edu (D.J.); david.cote@med.usc.edu (D.J.C.); gabriel.zada@med.usc.edu (G.Z.)

**Keywords:** endoscopic endonasal surgery (EES), anterior skull base meningiomas, patient selection, complication avoidance

## Abstract

Endoscopic endonasal surgery (EES) has fundamentally changed the management of anterior skull base meningiomas by enabling access to many lesions through a direct minimally invasive corridor that avoids traditional transcranial exposure. Improvements in endoscopic optical technology, surgical instrumentation, and skull base reconstruction have steadily expanded the range of tumors that can be treated through an endonasal approach. Even so, EES is not appropriate for every lesion, making thoughtful patient selection and surgical team experience the most important determinants of surgical success. This review explores where EES fits in the contemporary management of anterior skull base meningiomas, including olfactory groove, tuberculum sellae, and planum sphenoidale meningiomas, with particular attention to its indications, limitations, and situations in which EES may not be appropriate. Evidence comparing endoscopic endonasal and transcranial approaches for anterior skull base meningiomas is reviewed, with attention to how tumor anatomy, oncologic objectives, and individual patient characteristics influence the choice of approach. A major consideration is how surgical morbidity can be minimized without compromising oncologic and safety outcomes. Current strategies to protect critical neurovascular structures, reduce the risk of cerebrospinal fluid leakage, and improve skull base durability, including advances in vascularized reconstruction techniques, are discussed. Emerging outcome data and the evolving learning curve associated with EES are also reviewed. Rather than presenting EES as a universal solution, the evidence points toward a patient-centered approach in which operative strategy and clinical decision making is guided by tumor characteristics, anatomy, and surgical goals.

## Simple Summary

Accessing anterior skull base meningiomas is challenging because of the complex anatomy and the critical structures that surround this region. While traditional transcranial approaches remain an important part of skull base surgery, advances in endoscopic endonasal techniques have established endoscopic endonasal surgery (EES) as an importantly minimally invasive alternative for appropriately selected lesions. The benefits of this approach, however, are closely tied to appropriate patient selection, as the tumor’s size, location, and relationship with surrounding structures ultimately determine whether EES is a safe and effective option. This review examines the current role of EES for anterior skull base meningiomas, while addressing its indications, limitations, approaches to reducing complications, and the factors that guide surgical decision-making.

## 1. Introduction

Anterior skull base meningiomas represent a diverse group of benign and malignant lesions that arise from, or involve, the region where the intracranial cavity meets the sinonasal tract [1,2]. Their management is uniquely challenging because this area contains critical neurovascular structures within a very limited surgical space [3]. The optic nerves, internal carotid arteries, pituitary gland, frontal lobes, olfactory tracts, and multiple cranial nerves often lie immediately adjacent to these tumors, meaning that even small differences in surgical strategy may have important implications for functional preservation [4,5]. Accordingly, the objective is not simply maximal tumor removal, but durable disease control while preserving neurological function and minimizing treatment-related morbidity [4,5].

**Figure 1:**
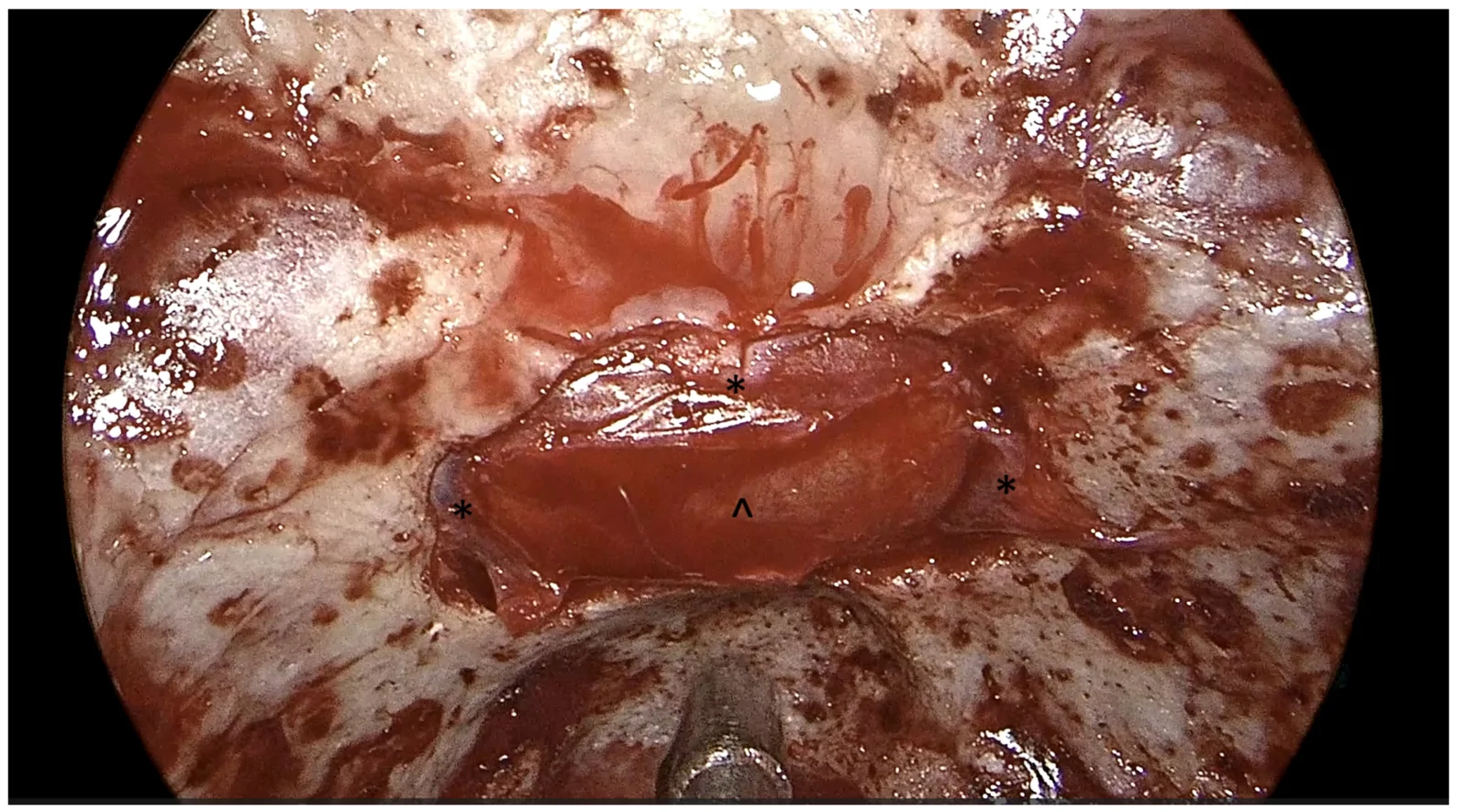
Endoscopic, transsphenoidal view of the pituitary gland during surgical resection of pituitary microadenoma causing Cushing’s disease. The image is not magnified. Asterisks indicate reflected dural leaflets exposing underlying normal pituitary gland, while caret indicates the underlying pituitary gland.

Historically, transcranial approaches were the standard surgical option for anterior skull base meningiomas. These operations provide wide exposure but typically require craniotomy, brain retraction, and more extensive soft tissue dissection [4,6]. These traditional invasive open approaches include a risk of retractor-related injury, cerebrospinal fluid leakage, pneumocephalus, and meningitis, among others. The introduction of endoscopic transsphenoidal surgery in the 1990s laid the foundation for the subsequent development of expanded endoscopic endonasal approaches over the next two decades, which significantly changed the management of selected anterior skull base meningiomas by providing a direct surgical corridor through the nasal cavity and paranasal sinuses [7,8]. Figure 1 displays one of our center’s images from transsphenoidal pituitary microadenoma surgery, showing the pituitary gland. Continuing advances in endoscopic optical technology, surgical instruments, neuronavigation, and skull base reconstruction techniques have enabled surgeons to treat an increasingly broad range of pathology through an expanded endonasal approach while maintaining favorable safety profiles and postoperative outcomes [9,10,11,12].

Even with these advances, the role of endoscopic endonasal surgery (EES) is still evolving rapidly. Favorable outcomes have been reported for many appropriately selected tumors; however, deciding whether an endonasal approach is appropriate depends on several factors, including tumor pathology, size, anatomical extent, involvement of nearby neurovascular structures, the experience of the surgical team, and to some extent patient preference [11,13]. For that reason, EES is best considered a complementary option for the skull base surgeon rather than a replacement for transcranial surgery, with the optimal application depending on patient selection [9,14]. This decision-making process is best undertaken by a multidisciplinary team that performs both open and endonasal approaches and deals with cases of equipoise frequently. This is especially true for anterior skull base meningiomas, where both endoscopic endonasal and transcranial approaches can achieve favorable results in carefully chosen patients, and the choice of surgical corridor remains an area of ongoing debate [9,11,15,16].

This review explores the current role of EES in the management of anterior skull base meningiomas, focusing on where these approaches offer the greatest benefit, where their limitations become apparent, and when they may not be the best surgical option. We also review the evidence comparing endoscopic endonasal and transcranial approaches for anterior skull base meningiomas and malignancies, examine current strategies for avoiding complications and optimizing skull base reconstruction, and discuss emerging technologies that continue to shape minimally invasive skull base surgery. Throughout, the emphasis is not on identifying a single preferred technique, but on guiding selection of the approach that best matches the tumor, the patient’s anatomy, and the goals of surgery.

## 2. Literature Search Strategy

This narrative review was developed from a focused review of the literature on EES for anterior skull base meningiomas. Relevant studies were identified in July 2026 through PubMed/MEDLINE and Google Scholar using search terms related to endoscopic endonasal surgery, anterior skull base meningiomas, transcranial surgery, skull base reconstruction, and CSF leaks. The years covered were 1990 to 2026. Rather than applying systematic review criteria, we sought to include studies that have had the greatest impact on modern skull base practice. Final papers were selected by giving priority to peer-reviewed original studies and landmark publications, while systematic reviews and meta-analyses were used to provide additional context when appropriate. The selected studies address patient selection, surgical outcomes, technical limitations, complication avoidance, reconstructive strategies, and recent advances in endoscopic skull base surgery. These publications form the basis of the discussion presented throughout this review.

## 3. Results and Discussion

### 3.1. Contemporary Role of Endoscopic Endonasal Surgery

**Figure 2:**
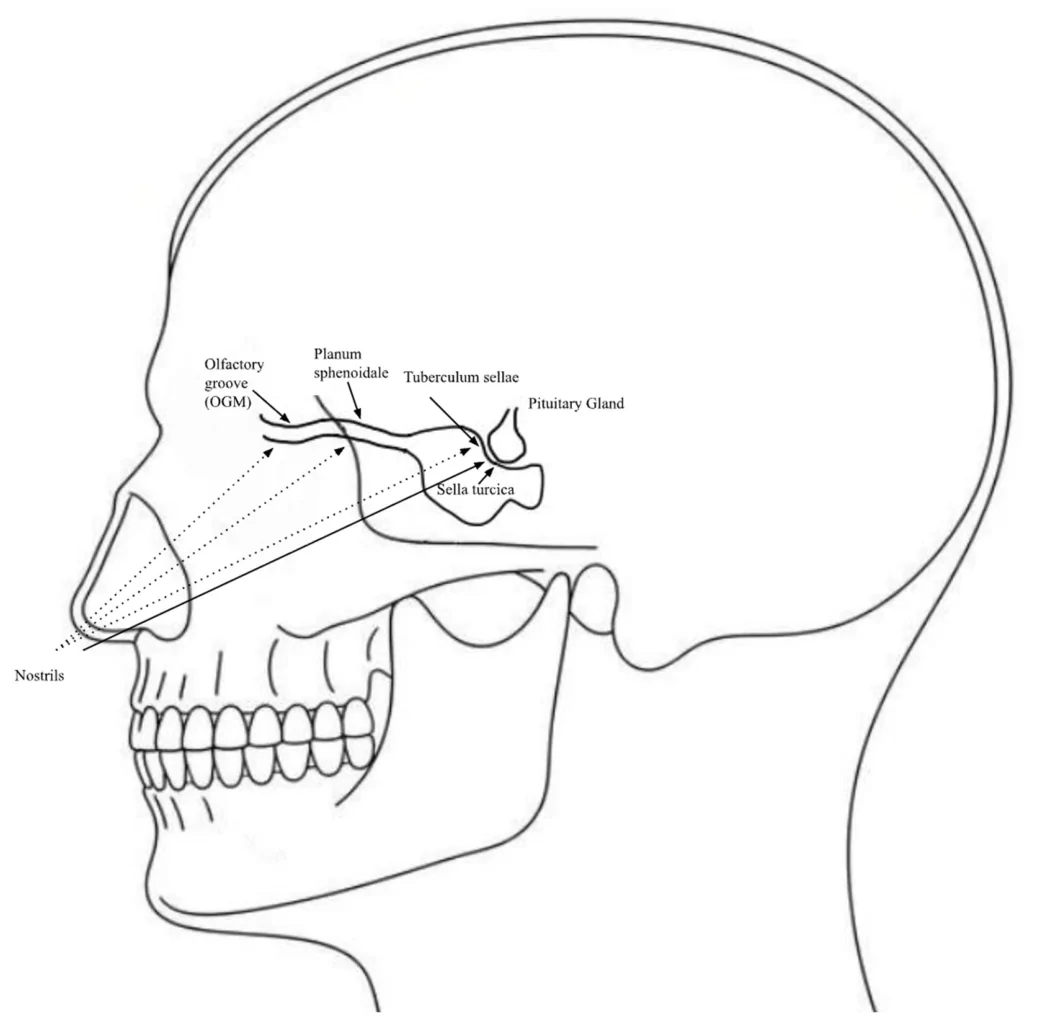
Expansion of the endoscopic endonasal surgical corridor to the anterior skull base. Early transsphenoidal approaches (solid line pathway from nostrils) initially provided access to sellar lesions, while subsequent expansion of the endoscopic endonasal approach (dotted line pathways from nostrils) enabled access to increasingly anterior skull base regions, including the tuberculum sellae, planum sphenoidale, and selected olfactory groove lesions. Advances in endoscopic visualization, instrumentation, and skull base reconstruction have enabled this expansion to anterior skull base meningiomas. OGMs, PSMs, and TSM are common anterior skull base meningioma subtypes arising along the midline cranial base [7,10,11,22].

The evolution of EES has substantially expanded the surgical options available for anterior skull base meningiomas. Initially developed for sellar lesions, EES has evolved into a versatile technique capable of treating increasingly complex pathology along the anterior cranial base [17,18]. Improvements in endoscopic visualization, specialized instrumentation, and skull base reconstruction have made this progression possible, allowing surgeons to access lesions that once required traditional transcranial exposure [10,19]. As a result, expanded endoscopic endonasal approaches have increased the number of tumors that can be treated through a minimally invasive corridor in carefully selected patients [17,20,21]. Figure 2 demonstrates a mid-sagittal anatomical schematic showing the expansion of the surgical corridor through EES.

Compared with traditional transcranial approaches, EES reaches the anterior skull base through a direct ventral corridor via the nasal cavity and paranasal sinuses, avoiding craniotomy and reducing the need for brain retraction [7,11]. This ventral trajectory often provides early exposure to the tumor attachment and vascular supply, especially in cases of meningiomas. It also facilitates progressive internal debulking while minimizing manipulation or retraction of adjacent neurovascular structures and brain tissue that may already be edematous. The endoscope also offers a panoramic, well-illuminated view of the operative field, and angled 30- and 45-degree lenses make it possible to visualize regions that are often difficult to see with a conventional operating microscope. These characteristics have made EES particularly attractive for midline lesions arising from the tuberculum sellae, planum sphenoidale, and selected olfactory groove meningiomas (OGM) [7,9,11,22]. It has also become paramount in the management of anterior skull base malignancies with the prototypical example being esthesioneuroblastoma, which arises in the cribriform region and can often be successfully resected via an extended transcribriform EES with flap reconstruction. This approach has also become the standard for spontaneous CSF leaks arising from the anterior skull base, often in the region of the fovea ethmoidalis in patients with idiopathic intracranial hypertension.

Recent advances in EES have been driven not only by improvements in endoscopic optics and surgical instrumentation but also by advances in image-guided navigation, high-speed drilling systems, intraoperative imaging, and vascularized skull base reconstruction. Specifically, reconstructive strategies have progressed from free tissue grafts and multilayer closure techniques to the routine use of vascularized flaps, particularly the pedicled nasoseptal flap [10]. This advancement has substantially reduced rates of postoperative CSF leakage, pneumocephalus and meningitis, which historically have been the major limitations of expanded endonasal surgery [10]. Improved reconstructive techniques have enabled safe treatment of larger and more complex lesions while preserving favorable neurological outcomes [23,24,25].

Although EES has expanded the range of lesions that can be treated through a minimally invasive corridor, it is not the right approach for every anterior skull base meningioma. Its greatest advantages are realized in carefully selected midline lesions, whereas tumors with marked lateral extension, extensive vascular encasement, dense calcification, or involvement of regions beyond the reach of the endonasal corridor (e.g., the frontal sinus) may be better managed through alternative approaches [9,15]. Expanded endonasal procedures also remain technically demanding, with outcomes influenced by surgeon experience and multidisciplinary collaboration with ENT [11]. As experience with both techniques has grown, the conversation has shifted away from asking which approach is better overall and toward identifying which approach is best suited for a given patient and pathology. This perspective underlies contemporary decision-making in anterior skull base surgery and provides the foundation for the following discussion of indications, limitations, comparative outcomes, and strategies for complication avoidance. Table 1 displays the features of tuberculum sellae, planum sphenoidale, and olfactory groove meningiomas that affect the choice between an endoscopic endonasal, transcranial, or combined approach.

### 3.2. Indications and Limitations of EES

#### 3.2.1. Indications

Appropriate patient selection remains the single most important determinant of successful outcomes following EES for anterior skull base meningiomas. Although advances in surgical technology have expanded the applicability of EES, the approach is not universally appropriate and should be individualized according to tumor pathology, anatomical location, extent of disease, involvement of surrounding neurovascular structures, and the goals of surgery [9,15]. Rather than relying on a single operative strategy, contemporary skull base surgery emphasizes matching the surgical corridor to the unique characteristics of each lesion in order to maximize tumor resection while minimizing operative morbidity [9,26].

In general, EES is best suited for meningiomas arising along the midline anterior skull base, where the endonasal corridor provides direct ventral access without the need for brain retraction [7,22]. This trajectory facilitates early identification of tumor attachment in cases of midline meningiomas, allows progressive internal debulking without early brain exposure or retraction, and provides excellent visualization of critical neurovascular structures using either a straight or angled endoscope [11,22]. Consequently, lesions centered on the tuberculum sellae, planum sphenoidale, and olfactory groove region have become among the most common indications for expanded endoscopic approaches, particularly when performed by a multidisciplinary skull base team [11,27].

Tuberculum sellae meningiomas (TSMs) have become one of the clearest indications for EES because their midline location is well suited to an endonasal approach [11,28]. From this trajectory, the optic canals and optic nerves can often be reached and decompressed early, before the bulk of the tumor is dissected, which may contribute to favorable visual outcomes following surgery [11,29]. In addition, this ventral approach also allows removal of associated hyperostotic bone and medial decompression of the optic nerves. In well-selected patients, EES has also been shown to achieve gross total resection rates similar to those of transcranial approaches while avoiding frontal lobe retraction and the morbidity that can accompany it [29,30,31,32]. Visual improvement and visual preservation rates have also been noted to be higher with EES, although selection bias is likely a major factor in some of the results noted from large systematic reviews and meta-analyses [9,15].

However, the appropriateness of EES for TSMs should not be determined by midline location alone. The direction and degree of optic canal invasion are important considerations, as EES provides direct access to the medial optic canal and is particularly advantageous when tumor involvement is primarily medial and can be decompressed through the endonasal corridor [28,29]. In contrast, extensive lateral optic canal involvement may be more difficult to address endonasally. Lateral tumor extension should also be considered in relation to the optic nerves and internal carotid arteries, as lesions extending substantially beyond these structures may limit the working angles and visualization available through the endonasal corridor [15]. Similarly, significant encasement of the internal carotid arteries, anterior cerebral arteries, or anterior communicating complex may increase the complexity of EES and favor a transcranial approach in selected cases. Preservation of an identifiable arachnoid plane between the tumor and surrounding neurovascular structures is another important consideration, as loss of this plane may increase the risk of vascular or neural injury during dissection [5]. Tumor consistency and calcification may further influence approach selection, with firm or heavily calcified tumors potentially being more difficult to mobilize and remove through the endonasal corridor. Finally, the relationship between the tumor and optic apparatus, including the direction of optic nerve displacement and degree of optic nerve compression, should be carefully evaluated preoperatively, as these anatomical features can determine whether the direct ventral trajectory of EES to TSMs provides a meaningful advantage for early optic canal and optic nerve decompression [11,28,29].

A similar principle applies to related PSMs. When these tumors remain largely confined to the midline and do not extend substantially lateral to the optic nerves or internal carotid arteries, they are often well suited for an expanded endoscopic endonasal approach [11,22]. The direct ventral trajectory allows the tumor origin to be identified and devascularized early, which can facilitate resection while limiting manipulation of the surrounding neurovascular structures [11,33]. As the tumor extends farther laterally or increases in size, however, the limitations of the endonasal corridor become more apparent, and alternative surgical approaches may provide safer or more complete resection [9,15].

The use of EES for OGM remains more selective compared with other midline anterior skull base meningiomas. In carefully chosen cases, particularly smaller or moderate-sized lesions that remain near the midline without significant lateral extension or extensive frontal lobe edema, an endoscopic approach can be considered at experienced skull base centers [11,34,35]. However, the anatomy of these tumors can quickly limit the feasibility of EES, especially when there is broad lateral attachment beyond the limit of the orbital roof, frontal sinus involvement, or significant vascular encasement [9,22]. Preservation of olfactory function should also be considered during surgical planning, particularly when evaluating patients with intact preoperative smell. For this reason, detailed preoperative evaluation of tumor extent and surrounding anatomy is critical when determining whether an endonasal corridor is appropriate, and in many cases these tumors may be better treated using a unilateral minimally invasive supraorbital or traditional subfrontal approach with olfactory nerve and tract preservation [15].

Similar principles of patient selection for EES apply to other skull base pathologies, including selected esthesioneuroblastomas, sinonasal malignancies with limited intracranial extension, craniopharyngiomas, and other midline lesions [36,37,38]. In each case, successful application of EES depends upon whether adequate oncologic resection can be achieved while preserving critical neurovascular structures and maintaining a durable skull base reconstruction [36,37]. In selected cases, a combined approach may be required, especially when frontal sinus involvement is present, or reconstruction is required using combination techniques including a pericranial flap and pedicled nasoseptal flap [10,39].

#### 3.2.2. Limitations

Despite its advantages, EES has limitations that must be considered before any surgical intervention. Perhaps the most important is the restricted lateral reach of the endonasal corridor. Tumors that extend well beyond the optic nerves, internal carotid arteries, or orbital roof can become increasingly difficult to access safely through this route [7,22]. Pushing the boundaries of the corridor in these cases may limit visualization, make dissection more technically demanding, and reduce safety and the likelihood of achieving a complete resection [9,11,15].

Tumor involvement of major neurovascular structures may also influence the suitability of EES. Tumors that completely encase the internal carotid arteries, anterior cerebral arteries, or other major vessels can be particularly challenging to dissect through an endonasal corridor and may carry a higher risk of vascular injury, which is challenging to manage via the endonasal approach [11,22]. The same is true when the tumor is densely adherent to the optic apparatus or small perforating vessels, or if superior–lateral optic canal involvement is present [9,11]. In these situations, a transcranial approach may provide better visualization and greater operative flexibility, allowing for a safer dissection [9,15]. Although increasing surgical experience and endoscopic visualization have expanded the ability to manage complex vascular anatomy through EES, surgeon expertise does not eliminate the anatomical risks associated with vessel encasement, perforator involvement, or loss of an arachnoid plane. These factors should therefore remain important considerations in approach selection, particularly because the options for managing major vascular injury differ between endonasal and transcranial approaches.

Not all of the limitations of EES are related to anatomy alone; the morphology of the tumor also matters. For example, heavily calcified or otherwise very firm tumors can be much more difficult to internally debulk through a narrow endonasal corridor, making resection more technically demanding [11]. More broadly, meningioma consistency can vary substantially, and firmer tumors requiring greater mechanical debulking may pose additional challenges when operating through a narrow surgical corridor [40]. The same is true for tumors that extend well into the frontal lobes or far laterally, where visualization and instrument mobility become increasingly limited. In these situations, achieving a gross total resection through an endonasal approach may be considerably more challenging and may not be in the best interest of the patient [9,15].

Patient-specific clinical and anatomical factors also play an important role in determining the optimal surgical approach. Large tumors causing substantial peritumoral edema, elevated intracranial pressure, or significant frontal lobe compression may require a wider operative exposure to achieve safe decompression [11,41]. Other factors, such as prior surgery, previous radiation, altered sinonasal anatomy, or chronic inflammatory disease, can make an endonasal approach technically more challenging and may influence both surgical access and reconstruction [10,42]. For these reasons, operative planning should always be individualized to the patient as well as the tumor [15].

Another major consideration when using EES is that the approach requires a high level of technical expertise and experience. The procedure involves more than simply accessing the tumor through the nasal corridor; it requires careful planning, collaboration between neurosurgeons and rhinologists, and reliable reconstruction of the skull base after resection [10,22]. Because of these demands, outcomes are closely tied to the experience of both the surgical team and the institution, as well as the associated learning curve [42,43,44,45]. Studies have shown that high-volume skull base centers often achieve better outcomes and fewer complications, emphasizing the importance of specialized teams when performing complex endoscopic procedures [42,44,45]. Patient preference has become an increasingly important factor, primarily in cases where surgical approach selection equipoise exists. 

### 3.3. EES Versus Transcranial Surgery for Anterior Skull Base Meningiomas

Anterior skull base meningiomas represent one of the most debated indications for EES. Unlike pituitary adenomas or many extradural skull base lesions, these tumors can often be approached safely through either an endoscopic endonasal or transcranial route. Consequently, the choice of surgical corridors has become less a question of technical feasibility and more one of optimizing tumor resection, preserving neurological function, minimizing surgical morbidity, and reducing long-term recurrence risk [9,11,15].

Transcranial approaches have decades of established clinical utility and continue to provide broad operative exposure, excellent lateral access, and flexibility for managing tumors involving major neurovascular structures or extending beyond the confines of the anterior skull base. Furthermore, minimally invasive craniotomy options exist (e.g., supraorbital eyebrow or mini-pterional) that may reduce operative time, reduce sinonasal morbidity, and provide excellent exposure of the tumor while maintaining principles of minimally invasive surgery and optimization of postoperative healing. Conversely, EES offers a direct ventral trajectory that avoids brain retraction, facilitates early devascularization of the tumor, and allows excellent visualization of the midline skull base through panoramic endoscopic optics [11,22]. As surgical experience and reconstructive techniques have improved, comparative studies have increasingly demonstrated that both approaches can achieve favorable outcomes when carefully matched to individual tumor anatomy and patient characteristics [9,15,32]. Because anterior skull base meningiomas comprise several anatomically distinct entities, comparison between surgical approaches is best considered according to tumor subtype and surgeon experience.

#### 3.3.1. Olfactory Groove Meningiomas

OGMs arise from the cribriform plate and frontoethmoidal region of the anterior cranial fossa and frequently present with headache, anosmia, cognitive changes, or visual disturbances resulting from progressive frontal lobe compression [46,47]. Their location places them among the most technically challenging anterior skull base meningiomas, as they often compress the unilateral or bilateral frontal lobes and may cause edema and altered mental status while remaining closely associated with the anterior cerebral arteries and surrounding neurovascular structures [11,48].

Traditionally, transcranial approaches have represented the preferred treatment for OGMs because they may be very large tumors with significant superior and/or lateral extension at the time of diagnosis, and open approaches provide broad visualization of both the tumor and adjacent vascular anatomy while permitting extensive lateral exposure. Approaches such as unilateral subfrontal, pterional, supraorbital, and bifrontal craniotomies allow surgeons to address tumors with significant lateral extension, extensive frontal lobe edema, vascular encasement, or involvement of the frontal sinuses while facilitating microsurgical dissection around critical neurovascular structures [46,47]. These characteristics continue to make transcranial surgery the preferred option for many large or anatomically complex OGMs [9,15] and may offer reduced operative times, CSF leak rates and sinonasal morbidity compared to expanded EES.

The application of EES to OGMs has expanded considerably over the past decade but remains more selective than for other anterior skull base meningiomas. The endonasal approach offers several advantages, including direct ventral access to the tumor attachment, early devascularization of the lesion, removal of involved bone and hyperostosis, avoidance of frontal lobe retraction, and reduced manipulation of the optic nerves [11,49]. Gardner et al., for example, showed that none of their thirty-five patients with OGMs experienced visual deterioration, whereas conventional open procedures for OGMs have rates of visual deterioration of about 20%, although elements of selection bias likely exist in these comparisons [11]. These benefits are particularly attractive for tumors centered along the midline with limited lateral extension and minimal surrounding edema [9,13,15].

On the other hand, tumors with broad lateral attachment beyond the orbital roofs, marked extension into the frontal lobes, extensive vascular encasement, or substantial peritumoral edema can be difficult to manage through the endonasal corridor because these features restrict visualization and increase operative complexity [9,50]. Another ongoing challenge is preserving olfactory function. Although postoperative anosmia remains common regardless of the surgical approach, rates of anosmia are higher with EES, as surgeons must go straight through the olfactory apparatus [51,52]. Therefore, preoperative olfactory function should be considered when selecting the surgical corridor. In patients with useful preoperative smell, unilateral supraorbital or frontolateral approaches may offer an advantage for olfactory preservation, whereas EES may be particularly attractive for patients who are already anosmic and have a midline OGM with hyperostotic cribriform involvement and limited lateral extension.

For many surgeons, transcranial surgery remains the preferred option for larger tumors or those with significant lateral extension, whereas EES is generally considered for carefully selected, smaller, midline lesions at experienced skull base centers [9,15,49,50].

#### 3.3.2. Tuberculum Sellae Meningiomas and Planum Sphenoidale Meningiomas

Among anterior skull base meningiomas, TSMs are widely considered one of the strongest indications for EES. Because these lesions arise in the midline immediately beneath the optic apparatus, they can often be reached directly through an endonasal corridor.

One of the principal advantages of EES for TSMs is the ability to visualize and decompress the optic canals and optic nerves early during surgery. Through an endonasal trajectory, surgeons frequently identify the medial optic canal before substantial tumor manipulation, allowing decompression of compressed neural structures prior to tumor mobilization [28,53,54,55]. This strategy has been proposed as one explanation for the favorable postoperative visual outcomes reported in numerous endoscopic series [32,56].

Visual preservation and visual improvement represent the most important functional outcomes in patients with TSMs. Multiple comparative studies have demonstrated excellent rates of postoperative visual improvement following both endoscopic and transcranial surgery, although several series suggest that EES may provide superior visual recovery in certain patients because of reduced optic nerve manipulation and earlier decompression [9,53,56]. Nevertheless, transcranial surgery remains an excellent option, particularly for tumors with substantial lateral extension, extensive optic canal involvement, or complex vascular anatomy that may limit safe endonasal dissection [57,58]. Transcranial surgery for anterior skull base meningioma may also offer several advantages, including faster operating room times, avoidance of sinonasal morbidity and CSF leaks, early visualization of the optic apparatus, and increased workspace. 

When comparing surgical approaches, the extent of resection remains one of the most frequently discussed outcomes. For a select-group of TSMs, studies have shown that EES can achieve gross total resection rates similar to those reported with transcranial surgery [28,59]. Although some series have found higher rates of complete resection with transcranial approaches, these differences are often seen in tumors with greater size or more complex extension [9]. Komotar et al. found that tumors in the open surgery cohort, which showed a higher rate of gross total resection, had a greater mean diameter than the EES cohort [9]. As a result, the variation in outcomes may be driven more by differences in tumor anatomy and case selection than by an inherent advantage of either approach [15,60,61].

PSMs occupy an intermediate position within the spectrum of anterior skull base meningiomas, and many surgeons view TSMs and PSMs as one continuum of a single pathological entity. Like TSMs, they often arise along the midline and may therefore be accessible through an endoscopic endonasal corridor. However, their tendency to extend superiorly and laterally with increasing size frequently introduces anatomical considerations that may challenge surgical planning [32,62].

Published outcomes indicate that both operative approaches can achieve favorable rates of gross total resection and neurological preservation when appropriately selected. For example, Agosti et al. reported a gross total resection rate of 80.9% for EES and 79.8% for transcranial approaches, and Hafiz et al. found gross total resection rates ranging from 82% to 96% for EES and 86% to 100% for transcranial approaches [28,63]. These findings further emphasize that surgical success depends less on the operative corridor itself than on appropriate patient selection [9,11,15].

#### 3.3.3. Comparative Outcomes and Limitations of the Evidence

To summarize the previous results, both EES and transcranial approaches can provide favorable outcomes for appropriately selected anterior skull base meningiomas, although reported results vary according to tumor location, size, extension, and patient selection. Published data demonstrate comparable gross total resection rates in selected tumors, for example, the previously mentioned results by Agosti et al. and Hafiz et al. [28,63]. For TSMs, EES may offer favorable visual outcomes because of early optic canal decompression and reduced manipulation of the optic apparatus, while transcranial approaches provide greater flexibility for tumors with substantial lateral or superior extension, vascular encasement, or complex neurovascular relationships [9,15,53,56,57,58]. EES is associated with a greater risk of postoperative CSF leak and, particularly for OGMs, loss of olfactory function due to the transnasal trajectory [51,52]. Vascular complications and recurrence have been reported with both approaches. Although transcranial surgery may provide better visualization and operative flexibility for tumors involving major vessels, direct comparisons remain limited by differences in tumor characteristics and follow-up between cohorts [9,15,50,57,58]. 

From an oncological perspective, the extent of resection should be considered alongside long-term disease control rather than as an isolated endpoint. Although gross total resection remains an important goal when it can be achieved safely, deliberate subtotal resection may be appropriate when complete tumor removal would require unacceptable manipulation of the optic apparatus, major vessels, or perforating arteries. In selected patients, residual tumors can be monitored or treated with stereotactic radiosurgery or radiotherapy, providing an alternative strategy when the morbidity of aggressive resection may outweigh the potential benefit of complete removal. Thus, recurrence and long-term tumor control should be interpreted in the context of the extent of resection, residual tumor location and volume, adjuvant treatment, and duration of follow-up [4,5]. Comparisons of recurrence and disease control should also account for differences in follow-up duration, as shorter follow-up may underestimate late recurrences and make outcomes between studies difficult to compare directly.

Much of the comparative literature is retrospective and subject to selection bias, with smaller, midline tumors with limited lateral extension more likely to undergo EES, while larger tumors with substantial lateral extension, vascular encasement, edema, or other unfavorable anatomical features are more likely to undergo transcranial surgery. Therefore, apparent differences in resection, visual, or complication rates may reflect differences in tumor anatomy and case selection rather than the surgical approach itself [9,11,15].

### 3.4. Complication Avoidance

Although EES offers important advantages for selected anterior skull base meningiomas, these procedures present unique technical challenges that require substantial experience, a team approach, and meticulous operative planning and execution. The evolution of expanded endoscopic approaches has been accompanied by significant advances in complication avoidance, particularly in the prevention of postoperative CSF leakage and the protection of critical neurovascular structures. Many of the innovations that have broadened the applicability of EES, including vascularized skull base reconstruction, image-guided navigation, and multidisciplinary skull base surgery, have been driven by efforts to improve patient safety while preserving the benefits of minimally invasive surgery.

#### 3.4.1. Cerebrospinal Fluid Leak Prevention

Postoperative CSF leakage has long been one of the major challenges associated with expanded EES for anterior skull base meningiomas. Because these procedures often create a sizable skull base defect, they can leave a direct pathway, or fistula, between the intracranial space and the sinonasal tract, increasing the risk of CSF leak, meningitis, pneumocephalus, and the need for additional surgery [64,65]. As a result, preventing postoperative CSF leakage by adequately reconstructing the anterior skull base has become a central priority in contemporary skull base surgery and has driven many of the advances that have helped make EES more widely adopted [10,25].

The risk of postoperative CSF leakage is influenced by several factors: defect size, the degree of intraoperative CSF flow and communication with cisterns and/or ventricles, tumor location, prior surgery or radiation, elevated intracranial pressure, and individual patient anatomy [65,66]. These factors may also influence approach selection before surgery. In particular, anticipated defect size, the likelihood of a high-flow CSF leak, prior surgery or radiation, available reconstructive options, and sinonasal anatomy may make an endonasal approach favorable for a given patient. Reconstruction is often particularly challenging within certain anatomical constraints, as observed in olfactory groove, PSMs, and TSMs, where the resulting anterior skull base defects tend to be larger and often more anterior than those encountered during routine sellar surgery and remain exposed to continuous CSF pulsations [10,67]. For this reason, minimizing postoperative leaks begins with careful surgical teamwork and planning, and depends on meticulous reconstruction and attention to detail [10,65].

Over time, a number of techniques have been introduced to lower the risk of postoperative CSF leakage. Throughout the operation, surgeons aim to preserve as much healthy tissue as possible, avoid unnecessary dural or arachnoid disruption, minimize sinonasal morbidity and anosmia, and achieve a secure multilayer closure of the skull base defect [10,65,68]. The use of lumbar drains remains somewhat more controversial. While some studies have reported lower leak rates in patients with high-flow intraoperative leaks or large skull base defects, others have found similar outcomes without routine lumbar drainage. For example, Harris et al. reported no postoperative CSF leaks in their relatively small cohort of twenty-nine patients with tumors of the suprasellar region, tuberculum sella, or planum sphenoidale who underwent EES, suggesting that lumbar drains were not necessary [69]. Conversely, Zwagerman et al. found that the use of lumbar drains significantly reduced the rate of postoperative CSF leaks among patients with high-flow intraoperative CSF leaks undergoing EES of the skull base [70]. Because of these mixed findings, the decision to place a lumbar drain is typically made on a case-by-case basis, considering the intraoperative findings and the surgeon’s experience. Successful reconstruction is also influenced by the surgical learning curve, with lower postoperative CSF leak rates consistently reported as institutional experience and multidisciplinary skull base expertise increase [44]. Smith et al. found a statistically significant drop in the number of patients with CSF leaks in early versus late cases of EES for skull base surgery [45]. In our center, lumbar drains are used for 48–72 h at a rate of 5 cc/hr for most extended EESs with minimal morbidity or complications [71]. Importantly, this is an institutional practice, not a general recommendation. Overall, lumbar drainage may be useful in cases involving large dural defects and extensive arachnoid dissection, which are associated with high-flow CSF leaks, but are not necessary in every case [69,70].

##### Skull Base Reconstruction

Contemporary reconstruction typically employs a multilayer closure tailored to the size and location of the skull base defect as well as the presence of low- or high-flow CSF leakage. Common reconstructive materials may include autologous fat grafts, fascia lata, collagen matrix, synthetic dural substitutes, and vascularized tissue flaps, which are frequently combined to create a durable barrier between the intracranial cavity and the sinonasal tract [65,70]. Many surgeons are steering away from autografting when possible to reduce donor site morbidity and pain. Selection of reconstructive techniques is individualized according to the characteristics of the defect, tissue availability, and surgeon preference.

One of the most important advances in endoscopic skull base surgery has been the widespread use of the vascularized nasoseptal flap. Owing to its robust blood supply and ability to cover large skull base defects, it has become the preferred reconstructive method for many anterior skull base procedures [10,72]. Compared with the free-graft techniques used in earlier years, vascularized reconstruction has been associated with substantially lower postoperative CSF leak rates and has played a major role in expanding the indications for EES [10,73]. For example, Chandran et al. found that pedicled vascularized flaps lead to superior outcomes in large skull base tumors in patients undergoing endoscopic skull base repair for CSF rhinorrhea [73].

#### 3.4.2. Neurovascular Protection

The goal of EES extends beyond achieving gross total resection, as preservation of the surrounding neurovascular structures is equally essential to achieving a good outcome. The quality of life following any skull base surgery (endoscopic or open) is paramount. Anterior skull base meningiomas often grow in close proximity to, or partially encase, critical structures such as the optic nerves, optic chiasm, internal carotid arteries, anterior cerebral arteries, perforating vessels, and cranial nerves [11,32,42]. Accordingly, meticulous microsurgical dissection with preservation of arachnoid planes, perforating vessels, and the vascular supply to the optic apparatus is critical to minimizing ischemic, visual, and other neurological complications during or following tumor resection.

Imaging is frequently used by surgeons to understand the anatomical considerations before surgery. Beyond simply showing tumor size and location, preoperative MRI, CT, and vascular imaging provide insight into the structures that may complicate resection, including the optic canals, carotid arteries, surrounding bone, and adjacent neurovascular anatomy [74,75]. Features such as optic canal involvement, vessel encasement, calcification, hyperostosis, or anatomical variations can all influence the surgical strategy [9,15]. This information is critical when determining whether an endoscopic endonasal approach will provide adequate exposure or whether a transcranial route would offer a safer alternative.

Protecting the surrounding neurovascular structures necessitates meticulous microsurgical techniques and new operative technologies. High-definition endoscopic visualization, angled endoscopes, neuronavigation, micro-Doppler ultrasonography, specialized surgical instruments, and intraoperative neurophysiological monitoring all contribute to protecting the surrounding neurovascular structures [76,77,78]. However, these technologies are only part of the equation. Careful tumor debulking and dissection, early identification of vascular anatomy, and preservation of natural arachnoid planes remain critical steps in reducing unnecessary manipulation and preventing neurological injury [24,79].

## 4. Conclusions

Over the past several decades, EES has changed the way many anterior skull base meningiomas are approached, offering an endonasal route to lesions that were previously managed primarily through transcranial corridors. Improvements in endoscopic visualization, instrumentation, navigation, and reconstructive techniques have continued to expand the role of EES, allowing surgeons to achieve tumor control while preserving neurological function in appropriately selected patients. Despite these advances, EES remains a highly individualized approach, and favorable outcomes depend on careful patient selection, a thorough understanding of tumor anatomy, surgeon experience, and recognition of the limitations of the endonasal corridor.

Rather than replacing transcranial surgery, EES has become another important option within the modern skull base surgeon’s armamentarium. For selected midline lesions, including tuberculum sellae, planum sphenoidale, and OGMs, the endonasal approach can provide direct access to the tumor while limiting manipulation of the brain and surrounding structures. Ultimately, the choice of surgical approach is not determined by the technique itself, but by how well the chosen corridor fits the individual patient, the characteristics of the tumor, and the goals of resection.

## 5. Conclusions and Future Directions

Future advances in endoscopic skull base surgery are likely to come from improvements in the technologies that support surgical planning and intraoperative decision-making rather than changes to the endonasal approach itself. New developments in endoscopic optics, surgical instrumentation, intraoperative imaging, augmented and virtual reality guidance, robotic assistance, and artificial intelligence-based planning are already beginning to influence how these procedures are performed [80,81,82,83]. Although many of these tools have not yet been widely adopted, they may improve visualization, navigation, and operative precision, ultimately making complex skull base surgery safer and more reproducible [84,85]. It is also important to recognize that advances in medical therapy, stereotactic radiosurgery, immunotherapy, and other novel avenues may evolve rapidly and affect the role of surgical resection and the goals of tumor control.

Skull base reconstruction remains one of the main factors that has allowed EES to expand to increasingly complex tumors. Although modern closure techniques have greatly improved the reliability of these procedures, reconstruction is still one of the most technically challenging parts of surgery, particularly for large defects. Current efforts are focused on improving the materials and methods used for repair, including tissue-engineered grafts, patient-specific reconstruction strategies, and new vascularized flap techniques [10,86,87]. Future studies should directly compare reconstructive techniques across different defect sizes, locations, and intraoperative CSF flow grades, while identifying patient- and procedure-specific factors that predict postoperative CSF leakage, reconstruction failure, and the need for revision surgery [88,89].

Lastly, much of the existing literature comes from retrospective single-center studies, where differences in patient selection, tumor characteristics, operative techniques, and outcome reporting make direct comparisons difficult [90,91]. More robust evidence from prospective multicenter studies, standardized outcome reporting, and longer follow-up will be needed to better define the role of each approach. As this evidence continues to develop, surgical decision-making will likely become increasingly tailored to the individual patient, with approach selection based on tumor anatomy, clinical factors, and the available expertise rather than preference for a single technique [60,92].

## Figures and Tables

**Table 1:** Tumor- and anatomy-specific factors guiding selection of endoscopic endonasal, transcranial, or combined approaches for anterior skull base meningiomas.

Meningioma	Features Favoring EES	Features Favoring Transcranial	When a Combined Approach May Be Considered
Tuberculum sellae meningioma	Midline location; tumor centered beneath optic apparatus; medial optic canal involvement amenable to early endoscopic decompression; limited lateral extension; favorable vascular anatomy	Substantial lateral extension; complex vascular anatomy/encasement; extensive lateral optic canal involvement; anatomy limiting safe endonasal dissection; need for increased lateral workspace	Tumor with substantial midline and lateral extension in which neither corridor alone provides adequate access
Planum sphenoidale meningioma	Midline attachment; limited lateral extension; favorable neurovascular relationships; anatomy accessible through the endonasal corridor	Increasing superior/lateral extension; extensive lateral or orbital involvement; complex neurovascular anatomy; anatomy limiting safe endonasal access	Extensive tumors with both midline skull-base and lateral components requiring complementary exposure or reconstruction
Olfactory groove meningioma	Midline tumor; limited lateral extension; minimal frontal edema; preoperative anosmia; hyperostotic cribriform involvement amenable to endonasal removal	Broad lateral attachment beyond orbital roofs; substantial frontal extension; significant peritumoral edema; vascular encasement; useful preoperative olfaction when preservation is prioritized	Large/complex tumors with substantial midline and lateral extension or complex reconstruction requirements

## Data Availability

No new data were created or analyzed in this study. Data sharing is not applicable to this article.

## Abbreviations

The following abbreviations are used in this manuscript:

EES	Endoscopic endonasal surgery
OGM	Olfactory groove meningiomas
CSF	Cerebrospinal fluid
ENT	Ear, nose, and throat/Otolaryngology
TSM	Tuberculum sellae meningiomas
PSM	Planum sphenoidale meningiomas
